# Role of the Subtilisin-like Serine Protease CJPRB from *Cordyceps javanica* in Eliciting an Immune Response in *Hyphantria cunea*

**DOI:** 10.3390/ijms24044170

**Published:** 2023-02-20

**Authors:** Wenxiu Wang, Fengmao Chen

**Affiliations:** Collaborative Innovation Center of Sustainable Forestry in Southern China, College of Forestry, Nanjing Forestry University, Nanjing 210037, China

**Keywords:** subtilisin-like serine protease CJPRB, protective enzymes, immune defense-related genes, *Hyphantria cunea*, *Cordyceps javanica*

## Abstract

*Hyphantria cunea* is a globally distributed quarantine plant pest. In a previous study, the *Cordyceps javanica* strain BE01 with a strong pathogenic effect on *H. cunea* was identified, and overexpression of the subtilisin-like serine protease CJPRB of this strain was found to accelerate the death of *H. cunea* (previous research results). In this study, the active recombinant CJPRB protein was obtained through the *Pichia pastoris* expression system. It was found that CJPRB protein administration to *H. cunea* via infectation, feeding and injection was able to induce changes in protective enzymes, including superoxide dismutase (SOD), peroxidase (POD), catalase (CAT) and polyphenol oxidase (PPO), and the expression of immune defense-related genes in *H. cunea*. In particular, CJPRB protein injection induced a more rapid, widespread and intense immune response in *H. cunea* compared to the other two treatment methods. The results suggest that the CJPRB protein may play a role in eliciting a host immune response during infectation by *C. javanica*.

## 1. Introduction

Entomopathogenic fungi, as natural regulators of pest populations, have an advantage over most entomopathogens in that they can penetrate the insect body surface to infect insects without ingestion [1]. *Cordyceps* species are a group of globally distributed entomopathogenic fungi that are capable of infecting more than 40 species of insects in 8 orders, exhibiting a broad spectrum of insecticidal activity [2,3,4,5,6,7,8]. Among *Cordyceps* spp., *Cordyceps javanica* is capable of infecting insects of 12 genera in 3 orders [9]. The mortality rate of *Hyphantria cunea* larvae infected by the strain *C. javanica* BE01 isolated by us previously was over 85% at 1 × 10^8^ conidia/mL, indicating that this strain has good application prospects [10].

Typically, disruption of the insect cuticle enables successful infectation by entomopathogenic fungi. This process of cuticle destruction involves the following steps: conidial adhesion, germ tube formation, appressorium formation and penetration peg formation [11,12]. The appressoria release an array of hydrolytic enzymes for cuticle penetration and generate mechanical pressure that allows the fungus to pierce the insect cuticle through the penetration peg [13,14]. After the entomopathogenic fungus penetrates the cuticle and reaches the hemocoel, it sequesters insect nutrients, produces toxins and destroys the host cells, eventually killing the host [15]. Cuticle-degrading enzymes are important factors affecting the penetration ability and virulence of entomopathogenic fungi and include a series of hydrolytic enzymes, such as lipase, chitinase and protease [16,17]. Among them, the subtilisin-like serine protease Pr1 and trypsin-like serine protease Pr2 are the proteases most closely involved in the ability of entomopathogenic fungi to penetrate the cuticle [18]. Pr1 has been shown to be the main and most effective protease involved in stratum corneum penetration [19]. In addition, the Pr1 protein is able to activate a protein hydrolysis reaction cascade that converts pro-phenol oxidase to phenol oxidase, which triggers melanization in insects. Phenol oxidase produces some toxic molecules, such as quinones and oxygen radicals, which may lead to the death of the host insect [20].

Insects resist infectation by exogenous pathogens through natural immunity. The natural immune response of insects occurs mainly in the body wall, midgut and hemocoel. Protective enzymes (peroxidase (POD), catalase (CAT) and superoxide dismutase (SOD)) are commonly present in insects; these enzymes scavenge superoxide anion radicals and hydrogen peroxide produced by insects to protect the organism and cells from damage [21]. In addition, polyphenol oxidase (PPO), an important enzyme in insects, can defend insects against invasion by foreign pathogens and plays an important role in the immune system of insects [22]. Activated PPO catalyzes the formation of phenolic substances to melanin, which encapsulates exogenous fungi or is distributed near wounds, facilitating insect wound healing and the killing of invading pathogens [23]. However, excessive melanization can lead to insect mortality, so insects have evolved a variety of tools to negatively regulate the entire PPO pro-activation pathway. As one such tool, serine protease inhibitors (serpins) are able to regulate insect melanization by inhibiting the activation of phenol oxidation [24,25]. In addition, the insect innate immune response requires the participation of various serine proteases (SP) to ensure the transmission and expansion of the immune signal, and serpins can regulate the activity of SPs, on the one hand, to accelerate and strengthen the insect immune response and, on the other hand, to control the immune response to a certain extent to protect the insects themselves from harm [26].

In addition to the above insect immunity-related proteins, some defense-related proteins also play a role in the defense against disease agents. Chitin deacetylase (CDA) is a chitin-degrading enzyme that strictly catalyzes the deacetylation of chitin to form chitosan and plays an important role in the regulation of insect growth and development and immune response [27]. Studies have shown that the expression level of the *Hccdas* gene first increases and then decreases after *H. cunea* nuclear polyhedrosis virus (HcNPV) infection. The expression level of the *Hccdas* gene decreased significantly after administration of the Cry1Ab35 protein via feeding [28]. Moreover, antimicrobial peptides (AMPs) are important defense factors in insect humoral immunity and play an important role in the innate immune system of insects. According to the composition and molecular structural characteristics of amino acids, some scholars have divided AMPs into four categories: cecropins, defensins, glycine-rich AMPs and proline-rich AMPs [29,30,31]. Cecropins were among the first antibacterial peptides isolated and exhibit activity against Gram-negative and Gram-positive bacteria. Among them, cecropin A was found to also have antifungal activity. In addition, Shin discovered the new immune-related proteins HDD-1, HDD-13 and HDD-23 in *H. cunea*, among which HDD-1 and -13 were rapidly upregulated and continuously expressed after *Escherichia coli* injection, while HDD-23 was gradually upregulated and also constitutively expressed after injection [32].

Our previous results showed that *C. javanica* BE01 overexpressing the subtilisin-like serine protease CJPRB could accelerate *H. cunea* death [9]. Therefore, to further understand the role of the CJPRB protein in the pathogenesis of BE01, in this study, we investigated the changes in protective enzymes and the expression of immune defense-related genes in the larvae of *H. cunea* after infection and after feeding and injection with the CJPRB protein.

## 2. Results

### 2.1. CJPRB Protein Overexpression and Purification and Enzyme Activity Assay

**Figure 1:**
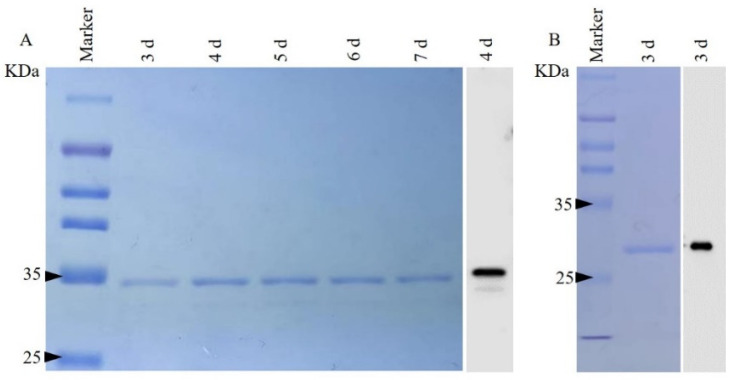
SDS–PAGE and Western blot analysis of the expression of recombinant CJPRB and GFP in *Pichia pastoris*. (**A**) SDS–PAGE and Western blot examination of purified recombinant CJPRB; (**B**) SDS–PAGE and Western blot examination of purified recombinant GFP.

The highest concentrations of the purified CJPRB protein (1166 μg/mL) and of purified GFP (1021 μg/mL) were obtained after 4 d of induction. Verification by SDS–PAGE showed that the size of the obtained CJPRB protein was 34 kDa and that the size of the GFP was 26 kDa (Figure 1). Moreover, the Western blot results confirmed that the purified proteins were recombinant CJPRB and GFP (Figure 1). In addition, the enzyme activity of CJPRB was determined to be 180.2 U/mL. The above results indicated that the recombinant CJPRB protein could be used in subsequent insect experiments.

### 2.2. Effects of CJPRB Protein Treatment on Larvae of H. cunea

**Figure 2:**
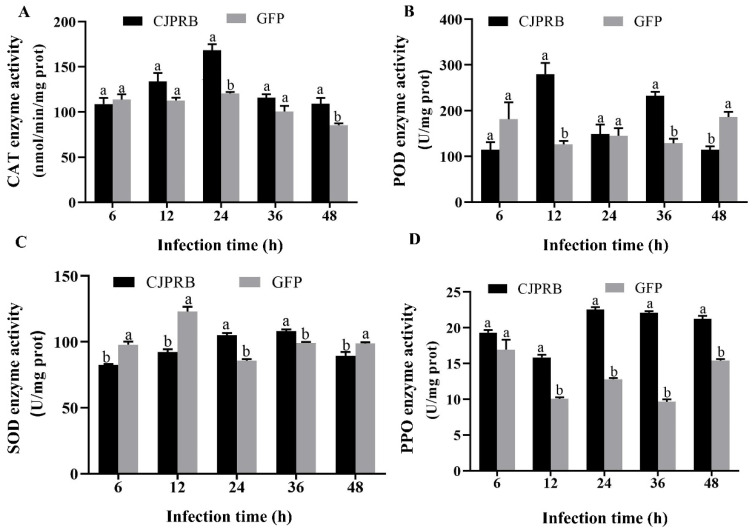
Changes in the activities of protective enzymes in *H. cunea* larvae after treatment with CJPRB and GFP. (**A**) Changes of catalase (CAT) in *H. cunea* larvae after infection with CJPRB and GFP for 6–48 h; (**B**) Changes of peroxidase (POD) in *H. cunea* larvae after infection with CJPRB and GFP for 6–48 h; (**C**) Changes of superoxide dismutase (SOD) in *H. cunea* larvae after infection with CJPRB and GFP for 6–48 h; (**D**) Changes of polyphenol oxidase (PPO) in *H. cunea* larvae after infection with CJPRB and GFP for 6–48 h. Means within a same time period followed by the different lower-case letter are significantly different (*p* < 0.05, Student’s *t* test).

The CAT enzyme activity at 24 and 48 h after CJPRB protein treatment of the second-instar larvae of *H. cunea* was significantly higher than that after the control GFP treatment, increasing by 39.58% and 27.68%, respectively (t = 7.049, df = 2.210, *p* < 0.05; t = 3.542, df = 4, *p* < 0.05) (Figure 2A). The POD enzymatic activity in the *H. cunea* larvae treated with CJPRB for 12 h and 36 h was significantly higher than that in the control GFP treatment group, increasing by 121.81% and 81.22%, respectively (t = 6.064, df = 4, *p* < 0.05; t = 7.991, df = 4, *p* < 0.05) (Figure 2B). In addition, the SOD enzymatic activity in *H. cunea* larvae treated with CJPRB for 24 h and 36 h was significantly higher than that in the control GFP treatment group, increasing by 22.40% and 9.06%, respectively (t = 9.810, df = 6, *p* < 0.05; t = 5.702, df = 6, *p* < 0.05) (Figure 2C). In particular, the PPO enzymatic activity in *H. cunea* larvae treated with CJPRB for 6 to 48 h was higher than that in the control GFP treatment group, and the enzyme activity of the group treated for 12 to 48 h was significantly different, increasing by 57.50%, 76.01%, 129.02% and 37.78%, respectively (t = 13.525, df = 6, *p* < 0.05; t = 27.413, df = 4.178, *p* < 0.05; t = 29.941, df = 6, *p* < 0.05; t = 12.572, df = 6, *p* < 0.05) (Figure 2D).

**Figure 3:**
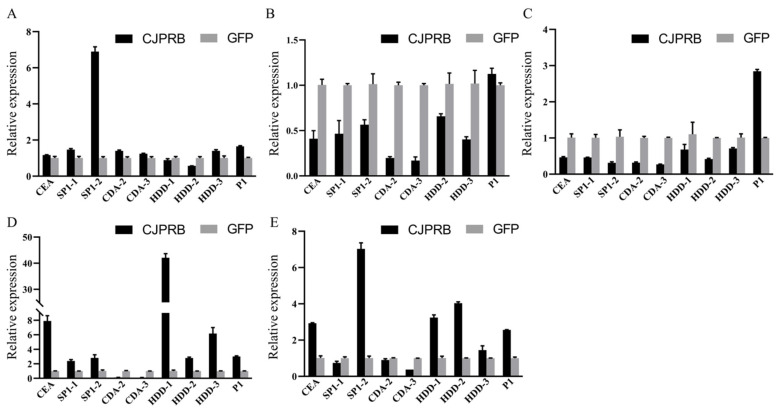
Expression of defense-related genes in *H. cunea* larvae 6–48 h after CJPRB treatment. (**A**) Expression of defense-related genes in *H. cunea* larvae 6 h after infection with CJPRB; (**B**) Expression of defense-related genes in *H. cunea* larvae 12 h after infection with CJPRB; (**C**) Expression of defense-related genes in *H. cunea* larvae 24 h after infection with CJPRB; (**D**) Expression of defense-related genes in *H. cunea* larvae 36 h after infection with CJPRB; (**E**) Expression of defense-related genes in *H. cunea* larvae 48 h after infection with CJPRB. GFP: control; CEA: cecropin A; SP1-1/-2: serine protease inhibitor; CDA-2/-3: chitin deacetylase; HDD-1/-2/-3: immune-related protein in *H. cunea*; P1: serine protease.

The results of RT–qPCR showed that, 6 h after infection, the expression of cecropin A (*CEA*), serine protease inhibitor (*SP1-1*/*-2*), chitin deacetylase (*CDA-2/-3*), immune-related protein (*HDD-3*) and serine protease (*P1*) was upregulated, and the expression of *SP1-2* was upregulated 6.90 ± 0.26-fold (Figure 3A). The expression of the serine protease *P1* in *H. cunea* was upregulated within 12–24 h of infection (Figure 3B,C). After 36 h of infection, the expression of cecropin A (*CEA*), serine protease inhibitor (*SP1-1*/*-2*), immune-related protein (*HDD-1/-2/-3*) and serine protease (*P1*) was upregulated, of which the expression of *HDD-1* was upregulated 42.11% ± 1.58-fold, and the expression of *CEA* and *HDD-3* was upregulated 7.90 ± 0.74- and 6.17 ± 0.82-fold, respectively (Figure 3D). After 48 h of infection, except for the serine protease inhibitor *SP1-1* and chitin deacetylase *CDA-2/-3*, all other defense-related genes were upregulated, and the expression of *SP1-2* was upregulated 7.03 ± 0.34-fold (Figure 3E).

### 2.3. Effects of CJPRB Protein Feeding on the Larvae of H. cunea

**Figure 4:**
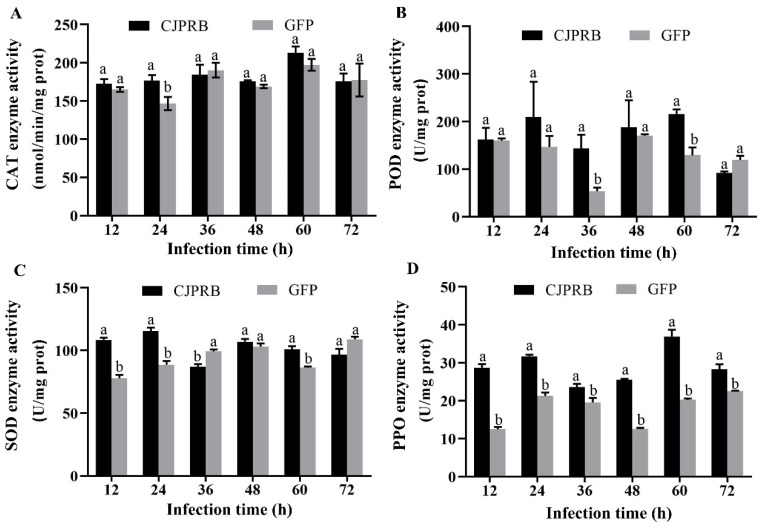
Changes in protective enzyme activity in *H. cunea* larvae after CJPRB and GFP protein feeding. (**A**) Changes of catalase (CAT) in *H. cunea* larvae 12–72 h after CJPRB and GFP feeding; (**B**) Changes of peroxidase (POD) in *H. cunea* larvae 12–72 h after CJPRB and GFP feeding; (**C**) Changes of superoxide dismutase (SOD) in *H. cunea* larvae 12–72 h after CJPRB and GFP feeding; (**D**) Changes of polyphenol oxidase (PPO) in *H. cunea* larvae 12–72 h after CJPRB and GFP feeding. Means within a same time period followed by the different lower-case letter are significantly different (*p* < 0.05, Student’s *t* test).

After the CJPRB protein was fed to the second-instar larvae of *H. cunea* for 24 h, the enzyme activity of CAT in *H. cunea* was found to be significantly higher than that in the control GFP treatment group, increasing by 19.79% (t = 2.887, df = 8, *p* < 0.05) (Figure 4A). POD activity in *H. cunea* fed CJPRB for 36 h and 60 h was not the same as that in the control GFP treatment group, and the difference was significant, with enzyme activities increasing by 168.33% and 67.22%, respectively (t = 3.045, df = 4, *p* < 0.05; t = 4.534, df = 4, *p* < 0.05) (Figure 4B). In addition, the SOD enzyme activity in *H. cunea* larvae fed CJPRB for 12, 24 and 60 h was significantly higher than that in the control GFP treatment group, increasing by 39.12%, 30.03% and 16.93%, respectively (t = 10.021, df = 6, *p* < 0.05; t = 6.633, df = 6, *p* < 0.05; t = 5.523, df = 6, *p* < 0.05) (Figure 4C). In particular, the PPO enzymatic activity PPO in *H. cunea* larvae fed CJPRB at various timepoints from 12 to 72 h was higher than that in the control GFP treatment group, increasing by 129.83%, 49.46%, 20.92%, 102.73%, 82.52% and 25.78%, respectively (t = 14.558, df = 8, *p* < 0.05; t = 10.428, df = 8, *p* < 0.05; t = 2.673, df = 8, *p* < 0.05; t = 32.840, df = 8, *p* < 0.05; t = 9.093, df = 4.379, *p* < 0.05; t = 4.283, df = 4.132, *p* < 0.05) (Figure 4D).

**Figure 5:**
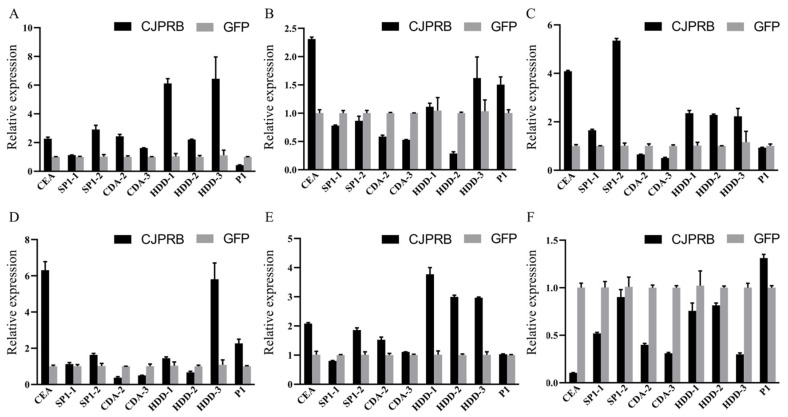
Expression of defense-related genes after CJPRB protein feeding in *H. cunea* larvae for 12–72 h. (**A**) Expression of defense-related genes in *H. cunea* larvae 12 h after CJPRB feeding; (**B**) Expression of defense-related genes in *H. cunea* larvae 24 h after CJPRB feeding; (**C**) Expression of defense-related genes in *H. cunea* larvae 36 h after CJPRB feeding; (**D**) Expression of defense-related genes in *H. cunea* larvae 48 h after CJPRB feeding; (**E**) Expression of defense-related genes in *H. cunea* larvae 60 h after CJPRB feeding; (**F**) Expression of defense-related genes in *H. cunea* larvae 72 h after CJPRB feeding. GFP: control; CEA: cecropin A; SP1-1/-2: serine protease inhibitor; CDA-2/-3: chitin deacetylase; HDD-1/-2/-3: immune-related protein in *H. cunea*; P1: serine protease.

The results of RT–qPCR showed that, after feeding for 12 h, expression of cecropin A (*CEA*), serine protease inhibitor (*SP1-2*), chitin deacetylase (*CDA-2/-3*) and immune-related protein (*HDD-1/-2/-3*) was upregulated and that of the immune-related protein *HDD-1/-3* was upregulated 6.12 ± 0.33- and 6.44 ± 1.52-fold, respectively (Figure 5A). After 24 h of feeding, the expression of cecropin A (*CEA*), immune-related protein (*HDD-1/-3*) and serine protease (*P1*) was upregulated (Figure 5B). After 36 h of feeding, the expression of cecropin A (*CEA*), serine protease inhibitor (*SP1-1*/*-2*) and immune-related protein (*HDD-1/-2/-3*) was upregulated, among which the expression of *CEA* was upregulated 4.09 ± 0.03-fold and that of *SP1-2* was upregulated 5.35 ± 0.09-fold (Figure 5C). After feeding for 48 h, except for the chitin deacetylase *CDA-2/-3* and the immune-related protein *HDD-3*, all other defense-related genes were upregulated (Figure 5D). Among them, *CEA* was upregulated 6.31 ± 0.47-fold, *HDD-3* was upregulated 5.81 ± 0.9-fold and *P1* was upregulated 2.27 ± 0.23-fold. After feeding for 60 h, except for the serine protease inhibitor *SP1-1* and serine protease *P1*, the other defense-related genes were upregulated, and the expression of *HDD-1/-2/-3* was upregulated 3.77 ± 0.23-fold, 3.00 ± 0.06-fold and 2.97 ± 0.03-fold, respectively (Figure 5E). After 72 h of feeding, only the serine protease *P1* was upregulated (Figure 5F).

### 2.4. Effects of CJPRB Protein Injection on H. cunea Larvae

**Figure 6:**
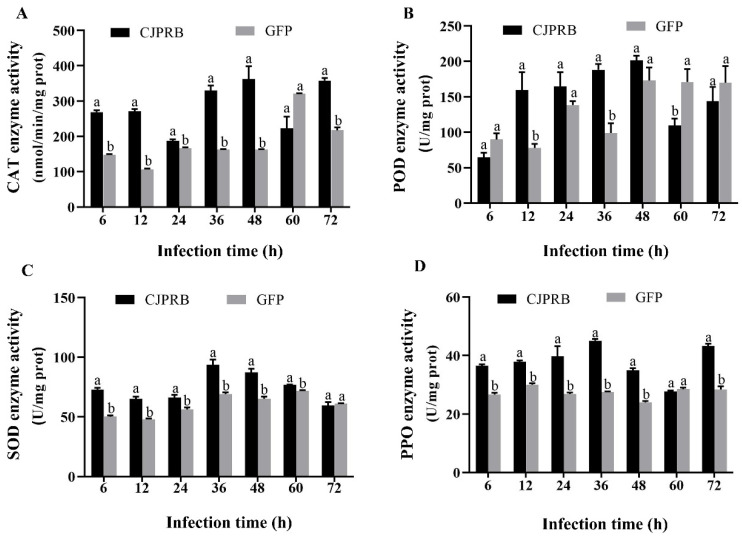
Changes in the activity of protective enzymes in *H. cunea* larvae after injection of CJPRB and GFP. (**A**) Changes of catalase (CAT) in *H. cunea* larvae after injection of CJPRB and GFP for 6–72 h; (**B**) Changes of peroxidase (POD) in *H. cunea* larvae after injection of CJPRB and GFP for 6–72 h; (**C**) Changes of superoxide dismutase (SOD) in *H. cunea* larvae after injection of CJPRB and GFP for 6–72 h; (**D**) Changes of polyphenol oxidase (PPO) in *H. cunea* larvae after injection of CJPRB and GFP for 6–72 h. Means within a same time period followed by the different lower-case letter are significantly different (*p* < 0.05, Student’s *t* test).

The results showed that after the CJPRB protein was injected into the fifth-instar larvae of *H. cunea* at various timepoints from 6 to 72 h, the CAT enzyme activity was significantly higher than that in the control GFP treatment group (except at 60 h), increasing by 81.86%, 154.96%, 12.57%, 103.25%, 123.43% and 64.75% (t = 21.188, df = 4.132, *p* < 0.05; t = 22.241, df = 4, *p* < 0.05; t = 4.05, df = 4, *p* < 0.05; t = 12.350, df = 2.031, *p* < 0.05; t = 5.590, df = 2.004, *p* < 0.05; t = 12.989, df = 4, *p* < 0.05) (Figure 6A). The PPO enzyme activity in *H. cunea* larvae treated with CJPRB for 12 h and 36 h was significantly higher than that in the control GFP treatment group, increasing by 104.50% and 90.12%, respectively (t = 3.182, df = 4, *p* < 0.05; t = 5.565, df = 4, *p* < 0.05) (Figure 6B) In addition, the SOD enzyme activity in *H. cunea* larvae injected with CJPRB at various timepoints from 12 to 60 h was significantly higher than that in the control GFP treatment group, increasing by 45.27%, 35.30%, 17.79%, 35.49%, 34.13% and 7.12%, respectively (t = 12.297, df = 6, *p* < 0.05; t = 8.126, df = 6, *p* < 0.05; t = 3.684, df = 6, *p* < 0.05; t = 5.087, df = 6, *p* < 0.05; t = 6.343, df = 6, *p* < 0.05; t = 7.953, df = 3.110, *p* < 0.05) (Figure 6C). In addition, after 6–72 h of CJPRB protein injection, except after 60 h of injection, the PPO enzymatic activity in *H. cunea* larvae was higher than that in the control GFP treatment group, increasing by 36.58%, 25.76%, 47.99%, 63.74%, 45.61% and 52.44% (t = 13.366, df = 8, *p* < 0.05; t = 12.143, df = 8, *p* < 0.05; t = 3.638, df = 8, *p* < 0.05; 0.05; t = 26.037, df = 5.034, *p* < 0.05; t = 13.137, df = 8, *p* < 0.05; t = 11.163, df = 8, *p* < 0.05) (Figure 6D).

**Figure 7:**
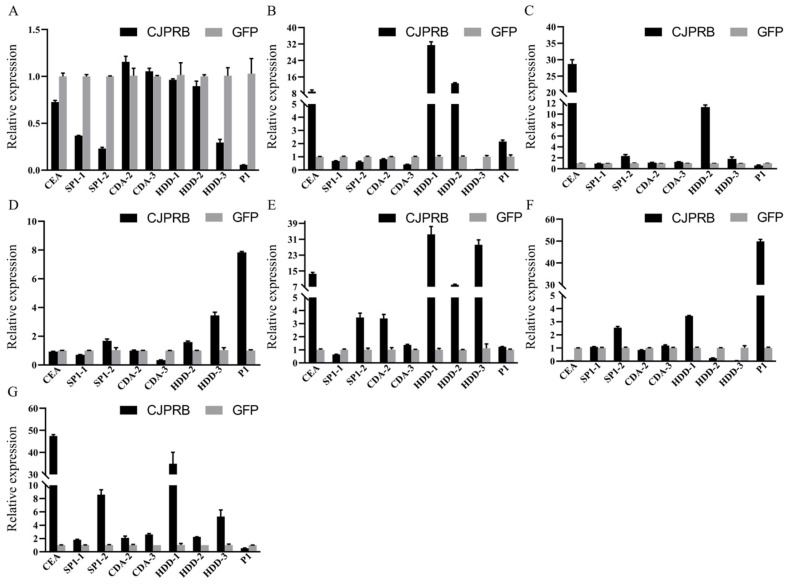
Expression of defense-related genes after injection of CJPRB in *H. cunea* larvae after 6–72 h. (**A**) Expression of defense-related genes after injection of CJPRB in *H. cunea* larvae after 6 h; (**B**) Expression of defense-related genes after injection of CJPRB in *H. cunea* larvae after 12 h; (**C**) Expression of defense-related genes after injection of CJPRB in *H. cunea* larvae after 24 h; (**D**) Expression of defense-related genes after injection of CJPRB in *H. cunea* larvae after 36 h; (**E**) Expression of defense-related genes after injection of CJPRB in *H. cunea* larvae after 48 h; (**F**) Expression of defense-related genes after injection of CJPRB in *H. cunea* larvae after 60 h; (**G**) Expression of defense-related genes after injection of CJPRB in *H. cunea* larvae after 72 h. GFP: control; CEA: cecropin A; SP1-1/-2: serine protease inhibitor; CDA-2/-3: chitin deacetylase; HDD-1/-2/-3: immune-related protein in *H. cunea*; P1: serine protease.

The results of RT–qPCR showed that the expression of chitin deacetylase (*CDA*-*2/-3*) was upregulated 6 h after CJPRB protein injection (Figure 7A). After 12 h of injection, the expression of cecropin A (*CEA*), immune-related protein (*HDD*-*1/-2*) and serine protease (*P1*) was upregulated 8.88 ± 0.84-fold, 31.47 ± 1.61-fold, 12.95 ± 0.28-fold and 2.15 ± 0.11-fold, respectively (Figure 7B). Twenty-four hours after injection, cecropin A (*CEA*), serine protease inhibitor (*SP1-2*) and immune-related protein (*HDD-2/-3*) were all upregulated, of which *CEA* was upregulated 28.67 ± 1.33-fold and *HDD-2* was upregulated 11.28 ± 0.38-fold (Figure 7C). In addition, 36 h after injection, the serine protease inhibitor (*SP1-2*), immune-related protein (*HDD-2/-3*) and serine protease (*P1*) were all upregulated, of which *HDD-3* was upregulated 3.44 ± 0.23-fold and *P1* was upregulated 7.83 ± 0.06-fold (Figure 7D). Forty-eight hours after injection, in addition to the serine protease inhibitor *SP1-1*, the expression of *CEA* was upregulated 13.63 ± 0.67-fold, and the expression of *HDD-1/-2/-3* was upregulated 33.49 ± 3.98-fold, 8.10 ± 0.23-fold and 28.23 ± 2.44-fold, respectively (Figure 7E). At 60 h after injection, the expression of the serine protease inhibitor *SP1-2*, immune-related protein *HDD-1* and serine protease *P1* was upregulated 2.55 ± 0.09-fold, 3.45 ± 0.04-fold and 49.86 ± 0.92-fold, respectively (Figure 7F). Finally, 72 h after injection, all the expression levels were upregulated, except for that of the serine protease *P1*, among which *CEA* was upregulated 47.45 ± 0.61-fold, *SP1-2* was upregulated 8.59 ± 0.74-fold, *HDD*-1 was upregulated 34.88 ± 5.20-fold and *HDD-3* was upregulated 5.28 ±1.01-fold (Figure 7G).

## 3. Discussion

In this study, we successfully expressed an active recombinant protein, CJPRB, through the *P. pastoris* expression system, and the protein was able to induce an immune defense response in larvae of *H. cunea* by infectation, feeding and injection.

The *P. pastoris* expression system is currently the most successful eukaryotic expression system, capable of post-translational processing of the expressed proteins and enabling better protein activity than that obtained via prokaryotic expression [33]. To date, more than 500 exogenous proteins have been successfully expressed in this eukaryotic expression system [34,35]. The activity of the Pr1A protein expressed by Zhang et al. through *P. pastoris* was 10.6-fold higher than that of the Pr1A protein expressed in a prokaryotic expression system by Wang et al. [34,36]. In the present study, active CJPRB protein was obtained by expression in *P. pastoris* and could be used in subsequent experiments.

The PPO system plays a key role in insect immune defense by recognizing foreign invading substances. In addition, the protective enzymes SOD, POD and CAT are present in a dynamic balance in insects, and this dynamic balance plays an important role in maintaining the metabolic balance in insects. However, when insects are under stress, the protective enzyme system in the body changes or even disrupts the normal dynamic balance, leading to metabolic disorders in insects. In CJPRB-treated larvae of *H. cunea*, POD was the first enzyme to respond to CJPRB treatment, followed by an increase in CAT and SOD enzyme activities, while the PPO enzyme activity associated with insect humoral immunity was significantly higher than the control within 12–48 h of CJPRB treatment. In contrast, after CJPRB feeding of *H. cunea* larvae, CAT was the first to initiate protective effects, and POD acted slightly later, while SOD acted in the early and late stages of treatment. The PPO enzyme activity was significantly higher than that in the control group throughout the treatment and continued to act during the observation period after feeding. After CJPRB injection of *H. cunea* larvae, CAT, SOD and PPO responded to CJPRB treatment at an early stage and continued to respond for almost the entire observed post-treatment period, while POD showed a significant increase in activity only at 12 h and 36 h post-injection. In conclusion, this study showed that the order in which the protective enzyme systems responded to CJPRB treatment of *H. cunea* larvae differed greatly. However, the PPO enzyme activity was significantly higher than that in the control group in the differently treated *H. cunea* larvae for almost the whole observation period, which also indicated that CJPRB was able to elicit an immune defense response in the insects and to sustain its action. The study by St. Leger and Gillespie also showed that the Pr1 protease activates PPO in insects and oxidizes phenols to quinones and that sustained oxidation leads to insect mortality [37,38].

In general, the clearance of invasive pathogens by host insects requires four steps: recognition of the foreign pathogen, initiation of an extracellular reaction cascade that activates serine proteases and disarms serine protease inhibitors, activation of downstream Toll or Imd signaling pathways and expression of different AMPs [39]. In the process of resistance to CJPRB protease treatment, the initial upregulated expression of serine protease inhibitor (SP1-2) and serine protease (p1) in *H. cunea*, followed by initiation of signal transduction via pathways inducing upregulation of the expression of the AMP CEA and immune-related proteins, is consistent with the whole process of insect defense against foreign invasion. However, SP1-2 was upregulated at both 6 and 48 h. This was, presumably, because CJPRB is a serine protease, so the upregulated expression of SP1-2 at 6 h may have been due to the recognition of the CJPRB protein; after 48 h, SP1-2 was upregulated again, to prevent the inhibition of serine proteases in *H. cunea* due to insect autoimmune overload.

The midgut is an important physical barrier against pathogenic infections in insects, and local production of AMPs is one of the main defense mechanisms in the insect midgut [40]. The expression of the AMP CEA in *H. cunea* was consistently upregulated during 12–48 h of feeding, because CJPRB induced the expression of AMPs in *H. cunea* midgut cells. The expression of the immune-related protein HDD in *H. cunea* was upregulated during 12–60 h of feeding. In addition, chitin deacetylase, which was not upregulated in the infectation experiment, was upregulated after 12 and 60 h of feeding, showing that CJPRB feeding can induce CDA-2 (CDA1) and CDA-3 (CDA2) expression in the intestine of *H. cunea*. It has been shown that CDA-2 (CDA1) and CDA-3 (CDA2) are mainly expressed in the brown moth epidermis, which is different from our results [27]. In addition, the expression of the serine protease P1 was not high during feeding. However, SP1-2 showed upregulated expression at all time points except at 48 h and 72 h, showing that CJPRB may activate the immune effect of other serine proteases in insects during the feeding treatment.

The expression levels of most immune-related genes after CJPRB protein injection were higher than those of immune-related genes after both the infectation and feeding treatments. Among them, the *H. cunea* immune-related protein HDD was upregulated and consistently expressed throughout the observed phase, which is similar to the pattern of expression of this class of genes in *H. cunea* observed after *E. coli* injection [32]. In addition to HDD, CEA, CDA, P1 and SP1-2 were also upregulated during most of the observation period after CJPRB injection, a result that also indicates that blood cavity injection of CJPRB can trigger a more rapid and widespread immune response in insects than other methods.

In conclusion, this study showed that the subtilisin-like serine protease CJPRB from *C. javanica* BE01 was able to induce an immune response in *H. cunea* by infectation, feeding and injection. However, the mechanism by which CJPRB induced the immune response through interactions with *H. cunea* is still unclear, and we will follow up with further studies on the interactions of CJPRB proteins in *H. cunea* to explore the contribution of CJPRB to the infectation of *H. cunea* by *C. javanica* BE01.

## 4. Materials and Methods

### 4.1. Strains, Plasmids and Insects

The *Pichia pastoris* KM71H and *E. coli* Top10 strains were stored in the Pathology Laboratory of Nanjing Forestry University.

The *P. pastoris* expression vectors pPICZαA and pPICZαA-GFP and the expression vector PYF11-CJPRB [9] (CJPRB GenBank number: OM468894) were stored in the Pathology Laboratory of Nanjing Forestry University.

Healthy 2nd- and 5th-instar larvae of *H. cunea* (the eggs were provided by the Chinese Academy of Forestry Sciences; after hatching, the larvae were incubated under 25 ± 2 °C and 65 ± 5% RH with a 12:12 h L:D photoperiod) reared in captivity were used for the CJPRB bioassay experiments.

### 4.2. Construction of the Expression Vector pPICZαA-CJPRB and Transformation of the P. pastoris Strain KM71H

The PYF11-CJPRB plasmid was used as a template to amplify the full-length coding sequence (CDS) of CJPRB (OM468894) using the primers CJPRBF (CCGGAATTCCGGATGCGCCTGTCCATCATCGCAGCAG) and CJPRBR (GCTCTAGAGCTCAATGATGATGATGATGATGATGATGAGTGGCGCCGTTGAAGGCCAGGTAG). The endonucleases EcoRI and Xbal were used to enzymatically cleave and linearize the vector pPICZαA. The pPICZαA vector was linearized by EcoRI and Xbal endonuclease. The CJPRB product obtained by PCR amplification and the linearized pPICZαA vector were recovered by using the EasyPure^®^ Quick Gel Extraction Kit (TransGen Biotech, Beijing, China). The recovered vector and CJPRB product were ligated overnight at 16 °C by using T4 ligase. The ligated product was transformed into competent *E. coli* Top10 cells, which were then coated on an LB selection plate containing kanamycin (50 µg/mL) and incubated overnight at 37 °C, after which the positive transformants were picked.

The pPICZαA-CJPRB plasmid was extracted from the positive transformants using the TIANprep Mini Plasmid Kit (TIANGEN, Beijing, China). Two micrograms of linearized pPICZαA-CJPRB (linearized by digestion with the endonuclease PmeI) was mixed with competent KM71H cells, and the mixture was transferred to a precooled 2 cm electrotransformation cup in an ice bath for 5 min. Electroconversion was performed using the following conditions: 1.5 kV voltage, 25 μV capacitance and 200 Ω resistance for 10 s. Immediately after electroconversion, 500 μL of prechilled 1 M sorbitol was added to the electrotransformation cup and mixed gently. The mixture was transferred to a sterilized centrifuge tube and shaken at 30 °C and 220 rpm for 1 h. The tube was centrifuged at 845× *g* for 3 min, and the cells were resuspended in a volume of 100 μL and spread uniformly on a YPD plate (containing 50 μg/mL bleomycin). The plate was incubated at 30 °C until a positive clone was produced.

### 4.3. Expression and Purification of the CJPRB Protein and GFP

A positive transformant (pPICZαA-CJPRB) was picked, added to 6 mL of liquid YPD medium (containing 50 μg/mL bleomycin) and incubated at 28 °C for 24 h with shaking at 200 rpm. One milliliter of the above-mentioned broth was inoculated into 240 mL of sterile BMGY medium in conical flasks (containing 30 mL of 1 M YNB); a total of 5 flasks were inoculated. After the culture was incubated for 24 h at 200 rpm and 28 °C, the supernatant was discarded, 120 mL of BMMY medium (containing 15 mL of 1 M YNB) was added to each of the 5 flasks and 1.2 mL of methanol was added to each flask to induce expression. Then 1.2 mL of methanol was added to each conical flask at regular intervals for 7 d at 200 rpm and 28 °C. On the 3rd, 4th, 5th, 6th and 7th days of methanol induction, one flask of bacterial culture was removed, and the supernatant was collected after centrifugation at 5432× *g* for 10 min. The centrifugation was repeated twice, and the final supernatant was collected.

GFP expression was induced as described above but only in one flask. Induction was performed by adding 1.2 mL of methanol per day, and the supernatant was collected after 3 d of induction (the induction conditions for GFP expression were determined in a previous study conducted in this laboratory).

CJPRB and GFPs were purified by Ni-NTA agarose purification resin preloaded onto gravity columns (Sheng Gong, Nanjing, China). The purified proteins (OD_280_ > 1) were placed in dialysis bags and left overnight at 4 °C in 1 M PB buffer. Protein concentration assays were performed using the Pierce™ BCA Protein Assay Kit (Thermo, Waltham, MA, USA).

### 4.4. SDS-PAGE and Western Blot Analysis

The purified proteins (4 μL) were visualized by 10% and 12% SDS-polyacrylamide gel electrophoresis (SDS-PAGE) and Thomas Brilliant Blue staining. For Western blot analysis, the recombinant CJPRB and GFPs were transferred to a polyvinylidene fluoride (PVDF) membrane at 200 mA for 2 h. The PVDF membrane was placed in 30 mL of blocking solution (1.2 g of skim milk powder dissolved in 1× PBST) and blocked at 4 °C overnight. The PVDF membrane was then removed, placed into 30 mL of blocking solution (1:5000) containing His-Tag (2A8) antibody (Abmart, Shanghai, China) and incubated on a low-speed shaker for 1.5 h. The PVDF membrane was removed, placed into 1× PBST, and washed for 10 min on a low-speed shaker; this step was repeated three times. The PVDF membrane was then placed in 30 mL of blocking solution containing goat anti-mouse IgG (1:10,000; Abmart, Shanghai, China) and incubated on a low-speed shaker for 0.5 h. The membrane was then placed in 1× PBST and washed on a low-speed shaker for 10 min; this step was repeated three times. Finally, the PVDF membrane was scanned and imaged with a dual-channel infrared fluorescence scanning imager.

### 4.5. CJPRB Protein Activity Assay

The activity of the subtilisin-like serine protease CJPRB was assayed by the Folin phenol method [41]. The tyrosine standard curve was obtained according to Zhuang’s method [41], with the equation y = 0.0047X + 0.00733, R^2^ = 0.9955. The OD_680_ value after the color development reaction was substituted into the standard curve equation to determine the concentration of tyrosine. Finally, the enzymatic activity of CJPRB was determined based on the tyrosine concentration. One unit of protease activity, expressed in U/mL, was defined as the amount of enzyme that produced 1 µg of tyrosine from the hydrolysis of casein in 1 min at 37 °C and pH 7.2.

### 4.6. CJPRB and GFP Infiltration of H. cunea Larvae

The 2nd-instar larvae of *H. cunea* were immersed in CJPRB (1 mg/mL) and GFP (1 mg/mL) solutions for 15 s, removed and placed on filter paper. Then the *H. cunea* larvae were placed into a rearing box after they had crawled and dried, and 0.1 g (approximately 30 heads) was sampled after 6 h, 12 h, 24 h, 36 h and 48 h.

### 4.7. CJPRB and GFP Feeding for H. cunea Larvae

The 2 × 2 × 2 cm square feed blocks were submerged in CJPRB (1 mg/mL) and GFP (1 mg/mL) protein solutions for 1 min and then removed after the feed had fully adsorbed the protein. The CJPRB- and GFP-treated feed was put into a feeder box and fed to starved *H. cunea* 2nd-instar larvae, and 0.1 g (approximately 30 heads) was sampled after 12 h, 24 h, 36 h, 48 h, 60 h and 72 h.

### 4.8. CJPRB and GFP Injection into H. cunea Larvae

Fifth-instar larvae of *H. cunea* (at the ventral segment of the third gastropod) were injected with 5 μL of protein per larva using a microsampler. Forty larvae were injected with CJPRB (1 mg/mL) and GFP (1 mg/mL) each, and 0.1 g was sampled after 6 h, 12 h, 24 h, 36 h, 48 h, 60 h and 72 h.

### 4.9. Activity Assay of Protective Enzymes in H. cunea Larvae after CJPRB Treatment

The Tissue and Cell Protein Extraction Kit (Epizyme, Shanghai, China) was used to extract total protein from CJPRB- and GFP-treated larvae of *H. cunea*. The concentrations of the extracted proteins were determined using the BCA Protein Content Assay Kit (Ke ming, Suzhou, China).

SOD, POD, CAT and PPO assay kits (Kemin, Suzhou, China) were used to determine the activities of these enzymes in *H. cunea* after CJPRB treatment. GFP-treated *H. cunea* was used as a control.

### 4.10. RT–qPCR Validation of the Expression of Defense-Related Genes in H. cunea after CJPRB Protein Treatment

RNA extraction from larvae of *H. cunea* and cDNA synthesis were performed according to Wang’s method [9]. The RT–qPCR system and conditions were as described by Wang [9]. The primers for the defense-related genes are shown in Table 1. The actin (*Hcactin*) gene of *H. cunea* was amplified as an internal control. The data were analyzed using the comparative threshold cycle (2−ΔΔCt) method. GFP-treated *H. cunea* was used as a control.

### 4.11. Statistical Analysis

Statistical analysis of the data and the significance of the differences between treatments were determined by Student’s *t* test. Statistical significance was set at *p* < 0.05.

## Figures and Tables

**Table 1:** RT–qPCR primers used to verify the expression of defense-related genes of *H. cunea*.

Gene (GenBank Number)	F	R
CEA (Cecropin A) (KJ660064)	GTGTTCGCTTGTTTCG	AGAACTTGAATAGCAGGAC
SPI-1 (serpin) (MH348864)	GAGTCAAGTGGAGGTGGTA	CATCTAAGAGTGTAGGGTCA
SPI-2 (serpin) (AF023278)	TGTATGTAAGTGACGCTGTA	AAAGACGAAGGGATGA
CDA-2 (CDA1) (KF975504)	AAACCCACAGGAAAGG	GTTATTGCCACCGACA
CDA-3 (CDA2) (KT781841)	TTGGACCAGTGGAAGC	AACACGCAGGTAGGGA
HDD-1 (HDD-1) (AF034998)	TCGGACAGGAAGATAA	ATGACAGCTTGCCACT
HDD-2 (HDD-13) (AF035000)	CCCATCGTCAACAAAGA	GCTCAGCCGTGTCAAA
HDD-3 (HDD-23) (AF035001)	ACTTCAGTTCCGACAA	CTTCAAATGATGGTGC
P1 (SP) (MH663425)	GCCCATAATCACCAAT	GTCAAGCCAACCAGTAG
HcActin (KT781843)	CTACCTCACGCCATTCTC	AGCTTCTCCTTGATGTCAC

## Data Availability

The datasets presented in this study can be found in online repositories. The names of the repositories and accession number(s) can be found at National Center for Biotechnology Information (NCBI) (https://www.ncbi.nlm.nih.gov/genbank/), OM468894, KJ660064, MH348864, AF023278, KF975504, KT781841, AF034998, AF035000, AF035001, MH663425 and KT781843.
